# Conned by the enemy: the entomopathogenic fungus *Metarhizium anisopliae* lures and kills *Drosophila suzukii*

**DOI:** 10.1002/ps.70576

**Published:** 2026-01-27

**Authors:** Ibrahim M Farid, Shepard Ndlela, Paul G Becher, Christopher W Weldon, Abdelmutalab GA Azrag, Helgi B Schiöth, Samira A Mohamed, Sunday Ekesi

**Affiliations:** ^1^ International Centre of Insect Physiology and Ecology (icipe) Nairobi Kenya; ^2^ Department of Zoology and Entomology University of Pretoria Pretoria South Africa; ^3^ Chemical Ecology‐Horticulture, Department of Plant Protection Biology Swedish University of Agricultural Sciences (SLU) Alnarp Sweden; ^4^ Department of Surgical Sciences Functional Pharmacology and Neuroscience, Uppsala University Uppsala Sweden

**Keywords:** biopesticide, virulence, horizontal transmission, horticulture

## Abstract

**BACKGROUND:**

*Drosophila suzukii*, commonly known as spotted wing drosophila (SWD), is a highly invasive and economically major pest that inflicts significant damage on soft‐skinned fruit crops, including raspberries, blueberries, strawberries, blackberries, cherries and grapes. The recent invasion of *D. suzukii* in Africa represents a key impediment to the berry industry on the continent. Conventional control strategies for this pest rely heavily on chemical insecticides, which pose several adverse side effects on biodiversity and environmental health. In this study, we evaluated five *Metarhizium anisopliae* isolates (ICIPE 7, ICIPE 18, ICIPE 20, ICIPE 30, ICIPE 78) for development as a biopesticide for *D. suzukii* management, by assessing both their direct pathogenicity and indirect effects via fungal volatile‐mediated behavioral responses.

**RESULT:**

All five *M. anisopliae* isolates led to high mortality in *D. suzukii.* The median lethal time (MLT_50_) showed that the ICIPE 78 isolate had the fastest action (4.75 ± 1.03 days) followed by ICIPE 7, ICIPE 18, ICIPE 30, and ICIPE 20, with MLT_50_ of 5 to 8 days. ICIPE 78 was horizontally transmitted by donor flies, and the fertility of recipient females was negatively impacted. We further documented that ICIPE 78 induced significant attraction to *D. suzukii*. Moreover, sporulated cadavers hosting ICIPE 78 attracted and infected healthy flies.

**CONCLUSION:**

The high pathogenicity of ICIPE 78 and its attraction to *D. suzukii* could be explored for pest suppression, especially as this isolate is already commercialized against other pests, which could facilitate its registration for use against *D. suzukii* through label extension. © 2026 The Author(s). *Pest Management Science* published by John Wiley & Sons Ltd on behalf of Society of Chemical Industry.

## Graphical abstract

Highly pathogenic isolates of *Metarhizium anisopliae* attracted *Drosophila suzukii*. Identifying the responsible compounds for this attraction could help the development of these isolates for pest monitoring and overall pest management.

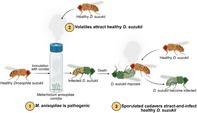

## 1 INTRODUCTION

*Drosophila suzukii* (Matsumura, 1931) (Diptera: Drosophilidae), also known as the spotted wing drosophila (SWD), is an important pest that causes serious damage to small soft‐skinned fruits such as raspberries, blueberries, strawberries, blackberries, and cherries, among others.^1^, ^2^ Native to Asia,^3^ the pest has spread to the Americas,^2^, ^4^ Europe,^1^ and most recently to Africa.^5^ The recent detection in Africa has sparked fears of devastating damage, which could disrupt the growing continental berry industry.

The most common management practice for this pest primarily relies on synthetic insecticides.^6^ Although insecticides are effective, their suppression of *D. suzukii* populations is often short‐lived and ineffective against larval stages, which are protected inside the fruit due to their cryptic life cycle.^7^, ^8^ Moreover, the constant use of insecticides poses several adverse effects on biodiversity including non‐target beneficial insects, the development of insecticide resistance, and environmental contamination, among others.^9^ Furthermore, postharvest intervals pose a great challenge to the timing of insecticide application, especially for horticultural products. This calls for alternative ecologically benign approaches to manage this pest, with various control methods under investigation and development.^6^ Among these, fungal‐based biopesticides are very promising and offer safer and more sustainable alternatives.

Fungi of the genus *Metarhizium* are common in the environment and have high insecticidal ability against several insect pests. For instance, *Metarhizium brunneum* negatively affects the survival of adult *D. suzukii* and also reduces fecundity premortem.^10^ Several Kenyan isolates of *M. anisopliae* such as ICIPE 7, ICIPE 18, ICIPE 20, ICIPE 30, ICIPE 69 and ICIPE 78 are highly pathogenic against numerous invasive horticultural pests including *Phthorimaea* (=*Tuta*) *absoluta* (Meyrick) (Lepidoptera: Gelichidae), *Spodoptera frugiperda* (J. E. Smith) (Lepidoptera: Noctuidae), *Bactrocera dorsalis* (Hendel) (Diptera: Tephritidae), and *Zeugodacus cucurbitae* (Coquillett) (Diptera: Tephritidae), with some isolates like *M. anisopliae* ICIPE 20 capable of targeting all of these pests.^11^, ^12^, ^13^, ^14^, ^15^, ^16^

Chemical attractants are used in attract‐and‐kill strategies to target certain pest species with no or minimal impact on non‐target organisms.^17^ The use of this approach in integrated pest management (IPM), enhances the precision of pest control efforts and thus promotes environmental sustainability.^18^, ^19^ The fungi *Beauveria bassiana* and *M. anisopliae* can attract insects like *Myzus persicae* (Sulzer) (Hemiptera: Aphididae) and *Anopheles stephensi* Liston (Diptera: Culicidae).^20^, ^21^ However, we are not aware of any reports regarding entomopathogenic fungi (EPF) attracting drosophilids. On the contrary, some fungal volatiles induce avoidance behavior in *Drosophila melanogaster* Meigen.^22^ Additionally, some insects can detect and actively evade EPF as natural enemies, and such avoidance behavior allows insects to minimize the risk of lethal infection.^23^ If *D. suzukii* evades EPF, this might jeopardize the efficacy of fungal‐based biopesticides, especially when utilized for autodissemination or in an attract‐and‐kill strategy.

Given these findings, we hypothesized that *M. anisopliae* isolates may infect and kill *D. suzukii* or influence its behavioral responses. To test this hypothesis, we assessed the pathogenicity of five Kenyan *M. anisopliae* isolates against *D. suzukii*. We also assessed *D. suzukii*'s behavioral responses to these fungal isolates. Our results demonstrate the potential of *M. anisopliae* for development into effective eco‐benign biopesticides against the invasive *D. suzukii*.

## 2 MATERIALS AND METHODS

### 2.1 Insect rearing

A *D. suzukii* colony was initiated in 2020 from flies obtained from field collected samples of berries on Longonot farm (S 00°50′21.2″, E 036°23′16.6″), Nakuru county, Kenya. Infested berries were placed in 2‐L plastic lunch boxes (82 mm × 156 mm × 220 mm), closed with fine nets to ensure proper ventilation and kept in the laboratory at the International Centre of Insect Physiology and Ecology (*icipe*), Nairobi, Kenya. In this study, emerged flies were separated into two colonies; one colony was maintained on an artificial diet for larvae while the other was maintained on store‐bought raspberries. Both colonies were kept under laboratory conditions, 21 ± 3 °C and 65% ± 5% relative humidity (RH) and a 12 h:12 h light/dark photocycle. Each laboratory population was maintained at a minimum of 500 individuals (with an approximate 1:1 sex ratio) and was supplemented biannually with a minimum of 50 wild‐caught flies. Prior to supplementation, wild flies were ice‐anesthetized for 10 min and examined under a stereomicroscope (ZEISS Stemi 508; ZEISS, Oberkochen, Germany) to exclude visibly injured or infected (e.g., mites or fungus grown) individuals.

For the colony maintained on raspberries, the fruits (VP FOOD company) were refrigerated at 4 °C for 48 h to kill potential insect contaminants. Consequently, seven raspberry fruits were placed on a Petri dish (90 mm × 15 mm) and transferred to a cage (20 cm × 20 cm × 20 cm) for rearing, containing 40 *D. suzukii* (1:1 sex ratio). To feed the flies, we used tephritid fruit fly hydrolysate yeast powder^24^ and provided a water‐soaked cotton ball in the cage.

The artificial diet was developed following the method described by Schlesener *et al*.^25^ with modifications. The ingredients comprised cornmeal (Formula 4‐24®, Carolina Biological Supply, Burlington, VT, USA), glucose (Excel Chemicals, Nairobi, Kenya), brewer's yeast LBI2240 (Lallemand, Montreal, Canada; currently not available), methyl paraben (Nipagin) (CAS: 99‐76‐3, Oxford Lab Fine Chem LLP, Mumbai, India), propionic acid (CAS: 79‐09‐4, Sigma‐Aldrich, St Louis, MO, USA), potato dextrose agar (PDA) (Oxoid Ltd, Basingstoke, UK), and distilled water (dH_2_O). First, a 10% stock solution of Nipagin was prepared by dissolving 0.8 g in 4 mL of absolute ethanol before adding 4 mL of dH_2_O. Then 0.8 g agar and 100 mL dH_2_O were heated in a microwave until the agar dissolved, after which it was poured into a container containing 8 g cornmeal, 10 g glucose, and 4 g yeast, and stirred until a homogeneous mixture was formed. Once the mixture cooled to 45 °C, 0.8 mL Nipagin from the stock solution and 0.3 mL propionic acid were added and mixed. The diet was poured into plastic Petri dishes (90 mm × 15 mm) to solidify, then transferred into a rearing cage as described earlier. The Petri dishes with cornmeal diet or raspberries were left in the cages holding flies for 72 h oviposition period, after which they were removed and incubated (21 ± 3 °C, 65% ± 5% RH, 12 h:12 h light/dark photoperiod) in a new cage until offspring emergence. For all the experiments 4‐ to 5‐day‐old flies were used.

### 2.2 Fungal culturing, suspension preparation and mass production

Twenty fungal isolates, including 15 *M. anisopliae*, one *M. brunneum*, two *B. bassiana* and two *Paecilomyces isaria*, were retrieved from *icipe*'s germplasm stock collection (storage in 10% glycerol at −80 °C). Thereafter, each isolate was separately sub‐cultured using 0.1 mL of the retrieved solution on 90 mm × 15 mm Petri dishes. *Metarhizium anisopliae* isolates were consistently cultured on Sabouraud dextrose agar (SDA) (Oxoid Ltd, Basingstoke, UK), while all other fungal species were cultured on PDA. The mother plates with the fungus culture were then incubated in the dark at 25 ± 2 °C for 14 days.

To test the viability of each fungal isolate, conidia from each mother plate were harvested and suspended in 10 mL of sterile 0.05% Triton X‐100 (CAS: 9036‐19‐5, Sigma‐Aldrich, St Louis, MO, USA) and vortexed for 3 min. From this initial suspension, a 1 × 10^6^ conidia/mL suspension was prepared through serial dilution, and conidial concentrations were determined using Neubauer's hemocytometer under a light microscope (Leica, UK) at 40× magnification. Then, 0.1 mL of the diluted suspension was plated onto new SDA or PDA plates and incubated for 18 h. The conidia growth on the new plates was stopped using lactophenol blue, and 100 stained germinated conidia were randomly counted with a hemocytometer (germination defined as germ tube ≥ 2× conidial diameter).^26^ The percentage of germination (PG) was determined using the formula: PG = (NGC/TNC) × 100, where NGC is the number of germinated conidia, while TNC is the total number of conidia observed. Once viability was ≥ 75%, for each isolate, the conidia on the mother plate were harvested to prepare suspensions of 1 × 10^8^ and 5 × 10^7^ conidia/mL, used for a screening bioassay against *D. suzukii* and fungal conidia mass production, respectively.

Based on the screening bioassay, isolates that caused ≥ 80% mortality were mass produced using Kenya Pishori milled rice (*Oryza sativa* L.) as a substrate^27^ to investigate their potential use as dry conidia against the pest. First, sterile liquid broth was prepared with 15 g bacteriological peptone (Oxoid Ltd, Basingstoke, UK), 30 g yeast extract (Oxoid Ltd, Basingstoke, UK), 30 g glucose, and 1000 mL dH_2_O. Then, 0.1 mL of fungal suspension (5 × 10^7^ conidia/mL) was added to 50 mL of liquid broth in a 250‐mL Erlenmeyer flask. This was kept at 25 ± 3 °C and 150 rpm for 3 days prior to inoculation into the rice substrate. Next, 2 kg of rice was washed with warm water and transferred into breathable biocontrol polypropylene bags (24 cm × 14 cm) each with a double B filter (Unicorn Imp. & Mfg. Corp, Plano, TX, USA), then autoclaved at 121 °C for 1 h. The bags containing rice, cooled to room temperature, were placed in a sterile laminar flow hood and inoculated with blastospores grown in liquid broth^28^ before the bags were sealed. The rice was gently massaged and incubated for 3 weeks at 20–26 °C and 40–70% RH. After incubation, the rice substrate with fungal conidia was left to dry for 5 days at room temperature. The conidia on the rice were then harvested by sieving the substrate through a sterile 295 μm mesh sieve and were transferred into sterile ziplock plastic bags (10 cm × 15 cm). Following a viability test, the ziplock bag of each isolate was stored at −80 °C for subsequent use.

### 2.3 Pathogenicity of fungal suspension and dry conidia against adult *D. suzukii*

A fungal suspension was used to screen the pathogenicity of each of the 20 isolates individually against diet‐reared *D. suzukii* adults. Flies were exposed to each fungal isolate in 90 mm × 15 mm Petri dishes lined with a Whatman filter paper (90 mm diameter) on both the base and lid. For each isolate, 6 mL of fungal suspension (1 × 10^8^ conidia/mL) was uniformly sprayed on the filter papers – 3 mL on the base and 3 mL on the lid. Twenty cold anesthetized flies (1:1 sex ratio; on ice for 10 min) were introduced into the base before the lid was secured for 3 min. This sample size followed the work of Toledo‐Hernández *et al*.,^29^ a commonly used standard in *D. suzukii* bioassays to balance feasibility and statistical power. Following exposure, flies were transferred into an experimental cage (15 cm × 15 cm × 20 cm) in a bioassay room at 21 ± 3 °C, 65% ± 5% RH and 12 h:12 h light/dark photoperiod. The control cage had 20 flies from a Petri dish sprayed with 0.05% Triton X‐100 (free of fungal conidia).

**Figure 1:**
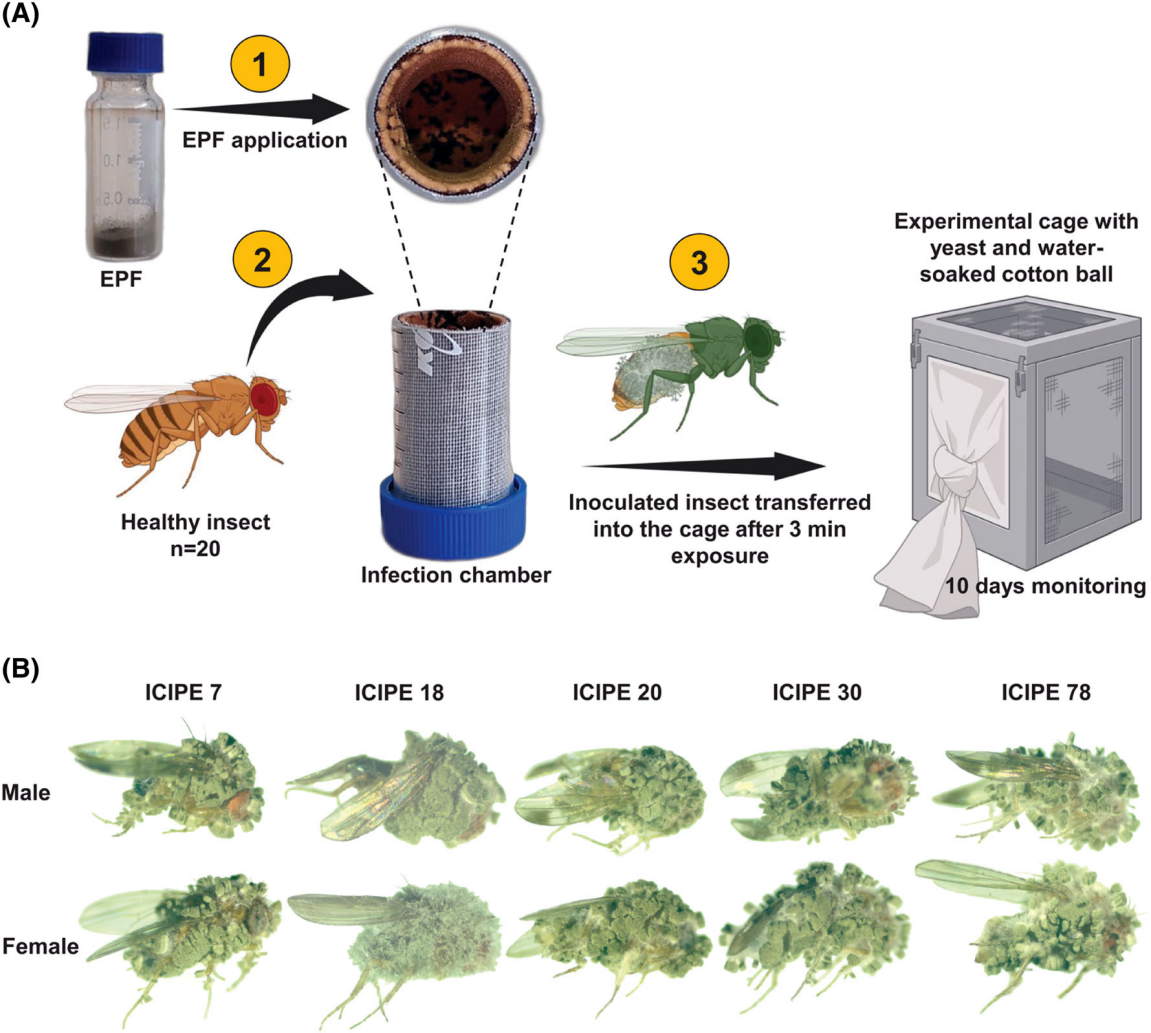
(A) *Drosophila suzukii* infection protocol and (B) sporulated *D. suzukii* after infection by fungal isolates of *Metarhizium anisopliae*. The images were captured 6–7 days after the death of infected *D. suzukii*.

The pathogenicity of dry conidia of five *M. anisopliae* isolates, ICIPE 7, ICIPE 18, ICIPE 20, ICIPE 30 and ICIPE 78, was tested against both diet and raspberry‐reared *D. suzukii* adults. Flies were exposed to the fungus using a cylindrical plastic chamber device (height 3 cm, diameter 2.5 cm) of which the inner walls were lined with sterilized velvet material (Fig. 1(A)). The latter was then smeared with 0.1 g of fungal conidia. Twenty flies (1:1 sex ratio) were then introduced into the chamber and allowed to walk and pick up the conidia for 3 min before being transferred into a cage in a bioassay room as described earlier. The control cage had 20 flies from a sterile infection chamber.

All experimental cages were supplied with yeast hydrolysate powder and a cotton ball soaked in dH_2_O to prevent fly mortality because of hunger or dehydration. The mortality of the flies was recorded daily for 10 days, and only for the bioassay for pathogenicity of dry conidia were the cadavers examined for mycosis. For each isolate, experiments were replicated three times.

### 2.4 Conidial acquisition and horizontal transmission by adult *D. suzukii*

Based on the dry conidia pathogenicity assay, *M. anisopliae* ICIPE 7 and ICIPE 78 were the most pathogenic against *D*. *suzukii*. Hence, they were subjected to further bioassays investigating fungal conidial acquisition and conspecific horizontal transmission between the flies. We used flies from the raspberry‐maintained colony in all subsequent trials to approximate, as closely as possible, the fitness of wild flies. Virgin flies (collected within 6 h of emergence and sex‐separated under cold anesthesia using a stereomicroscope) were used for the bioassay. Two cages, each containing 60 fungal free (non‐inoculated) flies were set up; one cage contained males (recipient‐males) and the second had females (recipient‐females). Subsequently, 60 female flies inoculated with fungal conidia (donor‐females) in the infection chamber were introduced to the recipient‐males' cage, while 60 similarly inoculated male flies (donor‐males) were added to the cage of recipient‐females. This setup was replicated once, providing sufficient flies for all bioassay sampling time points (0, 2, 4, 6, 8, 12, and 24 h). At 0 h (upon releasing donor‐flies into cages), a pair of flies (one male and one female) was randomly selected and collected from each cage. The collected flies were then individually introduced into different 15‐mL glass vials (9.5 cm × 2.85 cm) containing 1 mL of 0.05% Triton X‐100‐dH_2_O and glass beads. The vials were vortexed for 5 min to remove conidia from the flies, and the conidia from each fly were quantified using a hemocytometer.^27^ This procedure was repeated at 2, 4, 6, 8, 12 and 24 h. All selected flies were confirmed alive prior to vortexing. At each time point, six males and six females (*n* = 12) were sampled as independent biological replicates.

### 2.5 Effect of *M. anisopliae* dry conidia on the fertility of *D. suzukii*

*Metarhizium anisopliae* isolates ICIPE 7 and ICIPE 78 were tested for their effect on the fertility of *D. suzukii*. Newly emerged virgin flies were separated by sex and then exposed to the conidia of each fungal isolate (in a separate bioassay) in the infection chamber, after which they became donor‐flies, as previously described. Upon removal from the chamber, all flies engaged in grooming behavior for approximately 2 h. To accommodate this natural post‐infection behavior and reduce excess conidia, assays were conducted 4 h post‐infection. Pairs of *D. suzukii* (donors and recipients) were tested with three combinations: donor‐male + recipient‐female, donor‐female + recipient‐male, and donor‐male + donor‐female. The combination recipient‐male + recipient‐female served as the control. Each pair was tested in a glass vial (9.5 cm × 2.85 cm) containing a raspberry fruit, sealed with cotton to prevent flies from escaping. Pairs were transferred to new vials daily for ten consecutive days. Once the flies were transferred to the new vial, the vial with eggs was incubated under the same conditions described earlier and monitored daily for the emergence of new flies. The fertility of *D. suzukii* was obtained by determining the mean number of offspring emerging from the daily oviposition of parent flies. Over the 10 days, dead females in each vial were removed, and we considered that no offspring emerged for the following days. Dead recipient‐insects were incubated for 6–7 days at 25 ± 3 °C in sterile moist chambers (Petri dish lined with filter paper) and then examined (under stereomicroscope) for mycosis to confirm fungal transmission. For each fungal isolate, each combination used 12 couples (*n* = 12).

### 2.6 Behavioral response of *D. suzukii* to volatiles from dry conidia of *M. anisopliae*

The behavioral response of *D. suzukii* towards the five *M. anisopliae* isolates was tested in a dual choice attraction assay (one isolate at a time *versus* control). The testing arena consisted of a transparent polypropylene 4‐L plastic lunch box (17 cm × 10 cm × 15 cm). An opening was made in the center of the lid and covered with a fine mesh for ventilation (see figure 4(A)). For each isolate, 50 mg fungal conidia were put in a 2‐mL gas chromatography (GC) autosampler vial. The thin rubber disc of the GC vial cap was replaced with mesh to allow the release of fungal headspace volatiles. To avoid visual bias, the GC vial was wrapped in aluminum foil prior to being placed in a 50‐mL glass vial (9.5 cm × 2.85 cm). The latter was sealed with a customized plastic lid with a 4 mm‐diameter hole at the center, wide enough for the fly to pass. Another 50‐mL glass vial contained an empty GC vial wrapped in aluminum foil, which served as a control. The control and treatment vials were placed upright and 12 cm apart, at opposite ends of the experimental arena. A cotton ball soaked with dH_2_O and plugged into a 2‐mL centrifuge tube (dH_2_O reservoir) was provided. Thereafter, 20 flies (1:1 sex ratio; 24 h pre‐starved) were introduced to the arena before it was sealed. The number of insects inside the 50‐mL vials was counted after 2, 4, 6, 12, and 24 h. This bioassay was replicated eight times for each fungal isolate. Between replicates, the arena was cleaned with 70% ethanol and unscented detergent, then thoroughly rinsed and dried to eliminate residual volatiles and fecal matter. The attraction index (AI) was calculated using the formula: AI = ((NT − NC)/T) × 100, where NT is the number of flies in the treatment, NC is the number of flies in control, and T is the total number of flies.

### 2.7 Behavioral response of *D. suzukii* to *M. anisopliae*‐sporulated conspecific cadavers

This assay tested the behavior of flies when they encountered fungal conidia growing on a sporulated cadaver of a conspecific fly. The fungal isolates ICIPE 30 and ICIPE 78 were the most attractive based on the outcome of the flies behavioral response to the fungal isolates and hence, selected for this experiment. These two isolates were separately inoculated using the infection chamber to obtain sporulated *D. suzukii* cadavers. A sterile Petri dish served as the arena in this experiment. The lid of the Petri dish was lined with a Whatman filter paper (90 mm diameter) (see figure 6(A)) and placed on a bench. Then one sporulated cadaver was placed in the middle of the filter paper, and one cold anesthetized live *D. suzukii* was placed at 4.5 cm from the sporulated cadaver. To prevent flies from escaping, the Petri dish base (90 mm × 15 mm) was inverted onto the lid. Once the fly recovered from anesthesia, the insect was observed for 15 min, and its behavior was recorded. We considered attraction to have occurred if the introduced live *D. suzukii* was within 1 cm of the sporulated cadaver or in contact with it for a minimum duration of 1 min. Otherwise, we considered the fly avoided the fungal conidia. Fly choices were recorded for each isolate (*n* = 65 per isolate), using a new Petri dish with fresh filter paper for every replicate. Attracted flies were kept individually until death and then subjected to a mycosis test.

### 2.8 Statistical analysis

All data were analyzed using R statistical software (version 4.4.0),^30^ and the graphs were built using SigmaPlot v.14. The percentage of mortality (PM) of *D. suzukii* was adjusted with the control mortality using Abbott's correction.^31^ Adjusted mortality means were subjected to probit regression to estimate the median lethal time (MLT_50_) of the tested flies and the regression slopes using the *ecotox* package.^32^ The means of PG were analyzed using one‐way analysis of variance (ANOVA) with least significant difference (LSD) *post hoc* test while the means of PM and AI were analyzed using a generalized linear model (GLM) with a Gaussian distribution, followed by Tukey's *post hoc* test. The Kaplan–Meier estimator was used to estimate the daily mortality of *D. suzukii* over 10 days, and the survival curves of flies treated with each isolate were compared with those of non‐treated flies using the Log‐rank (Mantel–Cox) test. The data on conidia acquisition and horizontal transmission, as well as the data on the effect of the fungus on fertility, were not normally distributed. Therefore, the means were analyzed using the Kruskal–Wallis test with Dunn's *post hoc* test. The number of conidia retained in donor *versus* recipient‐flies after conidia transmission was compared using the Wilcoxon signed‐rank paired test. Chi‐squared (*χ* ^2^) tests were used to compare the sporulation rate in recipient‐flies after horizontal transmission, and the number of *D. suzukii* attracted to *versus* avoiding sporulated cadavers. The number of *D. suzukii* attracted to the fungal conidia was compared with the control using Wilcoxon paired test. All data were considered significant at *α* = 0.05.

## 3 RESULTS

### 3.1 Screening of fungal suspension pathogenicity against adult *D. suzukii*

The screening of 20 EPF isolates against *D. suzukii* adults revealed five *M. anisopliae* isolates (ICIPE 7, ICIPE 18, ICIPE 20, ICIPE 30 and ICIPE 78) causing significantly higher mortality to the flies compared with most other fungal isolates, regardless of species (*χ* ^2^(19) = 58 639, *P* < 0.001). However, the pathogenicity of *M. anisopliae* isolates ICIPE 7 and ICIPE 20 did not differ significantly from that of *M. anisopliae* isolates ICIPE 91 and ICIPE 69, or from *B. bassiana* isolate ICIPE 279 (Supporting Information Fig. S1).

### 3.2 Viability and pathogenicity of mass‐produced dry conidia against adult *D. suzukii*

Viability testing confirmed that mass‐produced dry conidia of all five *M. anisopliae* isolates, ICIPE 7, ICIPE 18, ICIPE 20, ICIPE 30 and ICIPE 78, were viable with over 93% germination. The germination rates differed among the isolates (*F*_4,10_ = 4.047, *P* = 0.033) with that of ICIPE 7 being the lowest compared to that of ICIPE 18, ICIPE 20, and ICIPE 78, but it did not significantly differ from that of ICIPE 30 (Table 1).

**Table 1:** Percentage of germination of mass‐produced dry conidia, and median lethal time (MLT_50_) and regression slope of *Metarhizium anisopliae* against *Drosophila suzukii* adults

Isolate	Rice substrate	Insects reared on artificial diet		Insects reared on raspberry fruits	
	% germination	MLT_50_ (days)	Slope	MLT_50_ (days)	Slope
ICIPE 7	93.92 ± 2.54b	4.95 ± 1.02	8.62 ± 0.66	6.51 ± 1.03	4.98 ± 0.42
ICIPE 18	99.80 ± 0.10a	6.08 ± 1.03	5.30 ± 0.42	8.67 ± 1.04	4.41 ± 0.47
ICIPE 20	99.42 ± 0.11a	8.04 ± 1.04	4.30 ± 0.43	9.85 ± 1.07	3.28 ± 0.39
ICIPE 30	96.28 ± 1.28ab	6.96 ± 1.02	6.71 ± 0.57	10.10 ± 1.06	4.33 ± 0.53
ICIPE 78	99.37 ± 0.31a	4.75 ± 1.03	7.19 ± 0.53	5.84 ± 1.02	6.72 ± 0.51

*Note*: Represented values are means ± standard error. Means followed by the same letter are not significantly different using *post hoc* Tukey least significant difference (LSD) test (*α* = 0.05).

**Figure 2:**
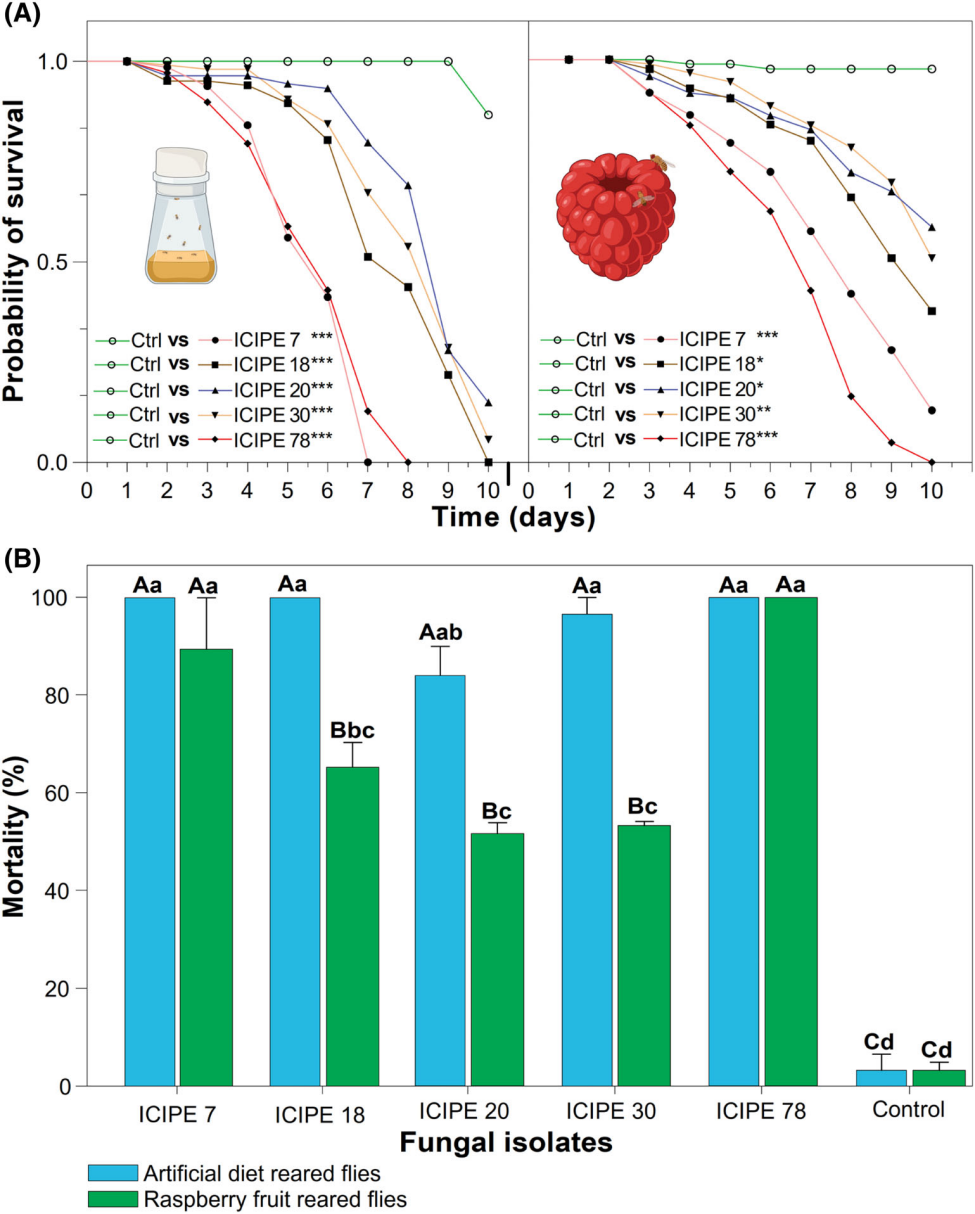
Efficacy of five *Metarhizium anisopliae* isolates on *Drosophila suzukii* adults. (A) Daily mortality curves of *D. suzukii* reared on artificial diet (left) and raspberry fruits (right). Each curve was compared to the control (Log‐rank, *α* = 0.05). (B) Cumulative percentage of mortality of *D. suzukii* after 10 days. Bars capped with the same lowercase and uppercase letters are not significantly different among fungal isolates and between rearing media within the same fungal isolate, respectively (Tukey's HSD test, *α* = 0.05).

The pathogenicity of dry conidia of these isolates was further evaluated against both diet and raspberry‐reared *D. suzukii* adults, resulting in visible mycosis in infected flies (Fig. 1(B)). The daily survival rates of *D. suzukii* reared on the artificial diet significantly decreased when exposed to fungal isolates compared to the control. A similar trend was recorded for the flies reared on raspberry fruit (*P* < 0.05; Fig. 2(A); Full statistics in Supporting Information Table S1).

Rearing media significantly influenced fly mortality, with flies reared on the artificial diet exhibiting significantly higher mortality compared to those reared on fruit when treated with ICIPE 18, ICIPE 20 and ICIPE 30 (*P* < 0.05; Fig. 2(B); full statistics in Table S2).

In terms of MLT_50_, ICIPE 78 isolate was the most virulent against flies regardless of whether the flies were reared on artificial diet or raspberry fruit. In contrast, ICIPE 20 and ICIPE 30 were the least virulent against diet‐reared and raspberry‐reared flies, respectively (Table 1).

### 3.3 Dry conidial acquisition and horizontal transmission by adult *D. suzukii*

At 0 h, *D*. *suzukii* acquired high numbers of conidia of ICIPE 7 and ICIPE 78 in the infection chamber, ranging from 17.01 ± 3.82 × 10^5^ to 20.38 ± 1.76 × 10^5^, and overall, these numbers gradually decreased over time (*H*(6) = 23.115, *P* < 0.001 and *H*(6) = 34.199, *P* < 0.001 for donor‐males and donor‐females, respectively, for ICIPE 7; and *H*(6) = 30.762, *P* < 0.001 and *H*(6) = 32.469, *P* < 0.001 for donor‐males and donor‐females, respectively, for ICIPE 78) (Fig. S2).

Regarding conidial transmission, *D. suzukii* transferred conidia to the opposite sex for both isolates (Fig. S2). For ICIPE 7, conidial loads retained by both donor sexes (males and females) were significantly higher than those retained by their recipients (females and males, respectively) at all time points except 24 h (*P* < 0.05; Fig. S2; full statistics in Table S3). However, for ICIPE 78, the number of conidia retained by donor‐males was comparable to that of their respective recipient‐females only at 4 and 12 h (*P* < 0.05). Conversely, the number of conidia retained by donor‐females was significantly higher than that retained by their respective recipient‐males at 2, 4, 6, 8 and 12 h (*P* < 0.05; Fig. S2; full statistics in Table S3).

### 3.4 Effect of *M. anisopliae* dry conidia on the fertility of *D. suzukii*

**Figure 3:**
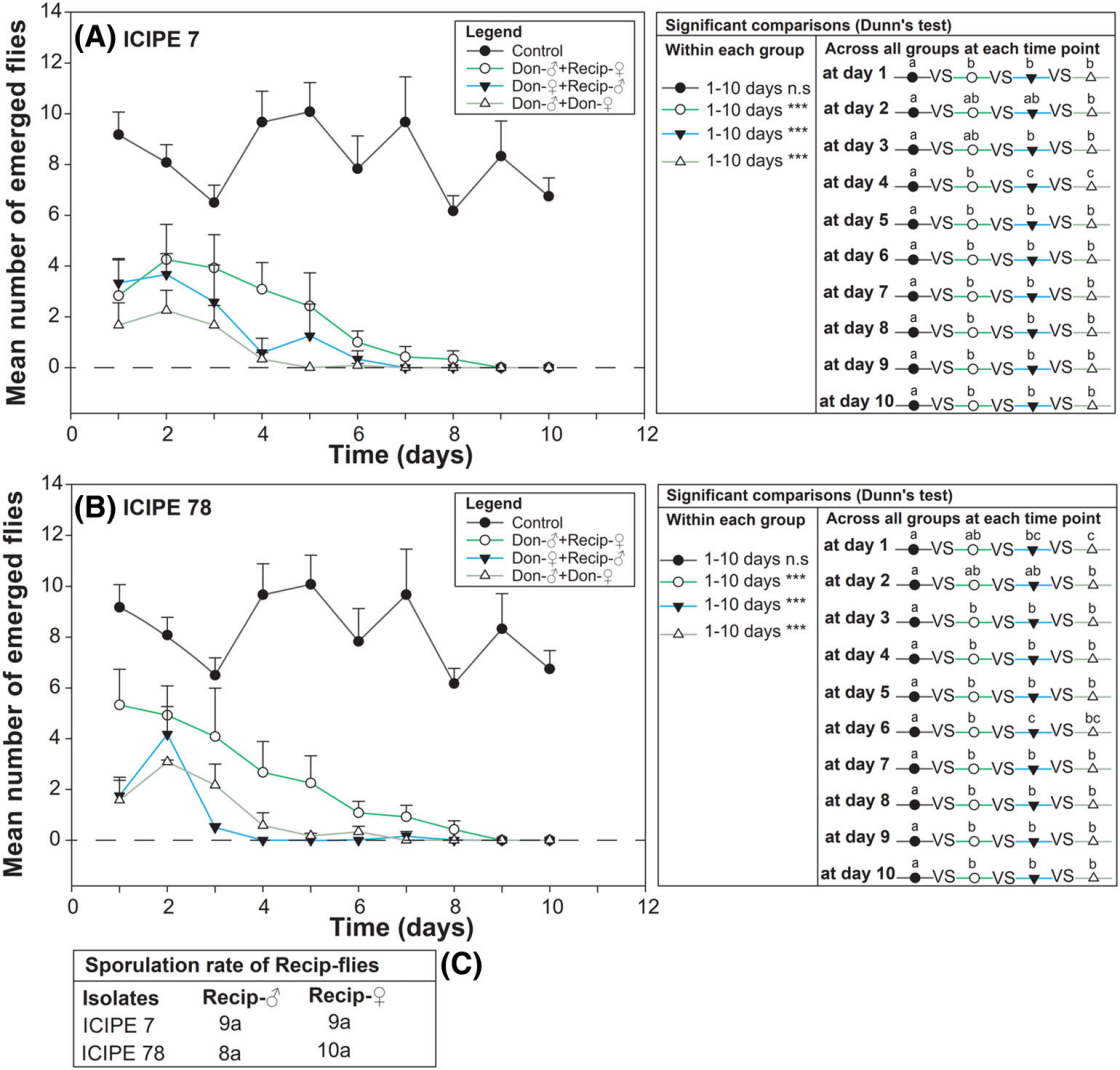
Mean number of offspring emerging from daily oviposition by infected and non‐infected fly parents (A,B). (C) Displays the number of sporulated recipient‐flies at the end of the bioassay. For each fungal isolate, the same lowercase letter and the asterisk symbol ‘*’ show significant differences among means or numbers (*post hoc* at *α* = 0.05; ***: *P* ≤ 0.001; n.s, not significant). Don., donor; Recip., recipient.

The most virulent isolates, ICIPE 7 and ICIPE 78, negatively impacted the fertility of *D. suzukii* (Fig. 3). The highest number of offspring emerged from healthy parents (control) on day 5, albeit this was not statistically different from other time points. Infection of either parental fly with ICIPE 7 or ICIPE 78 isolates led to significant fluctuations in daily offspring emergence over 10 days (*P* < 0.001 for ICIPE 7 and ICIPE 78, covering all combinations donor‐male + recipient‐female, donor‐female + recipient‐male and donor‐male + donor‐female) (Fig. 3(A),(B); full statistics in Table S4). The highest number of offspring from all *D. suzukii* pairs exposed to ICIPE 7 was recorded on day 2. In contrast, for ICIPE 78, the peak offspring production occurred on day 1 for donor‐male + recipient‐female pairs, whereas for donor‐female + recipient‐male, and donor‐male + donor‐female pairs, the highest number was recorded on day 2 (Fig. 3(A),(B)).

When comparing the number of offspring from treated parents to that of the untreated ones (control parents), infection by ICIPE 7 had a significant effect on fly emergence on all tested days (except for days 2 and 3) (*P* < 0.01, covering 1, 4–10 days). Similarly, except for days 1 and 2, ICIPE 78 caused a significant reduction in offspring emergence (*P* < 0.001, covering 3–10 days) (Fig. 3(A),(B); full statistics in Table S4).

Although most recipients showed evidence of mycosis, there was no significant difference in sporulation between recipient‐males and recipient‐females for either isolate (Fig. 3(C)).

### 3.5 Behavioral response of *D. suzukii* to volatiles from dry conidia of *M. anisopliae*

**Figure 4:**
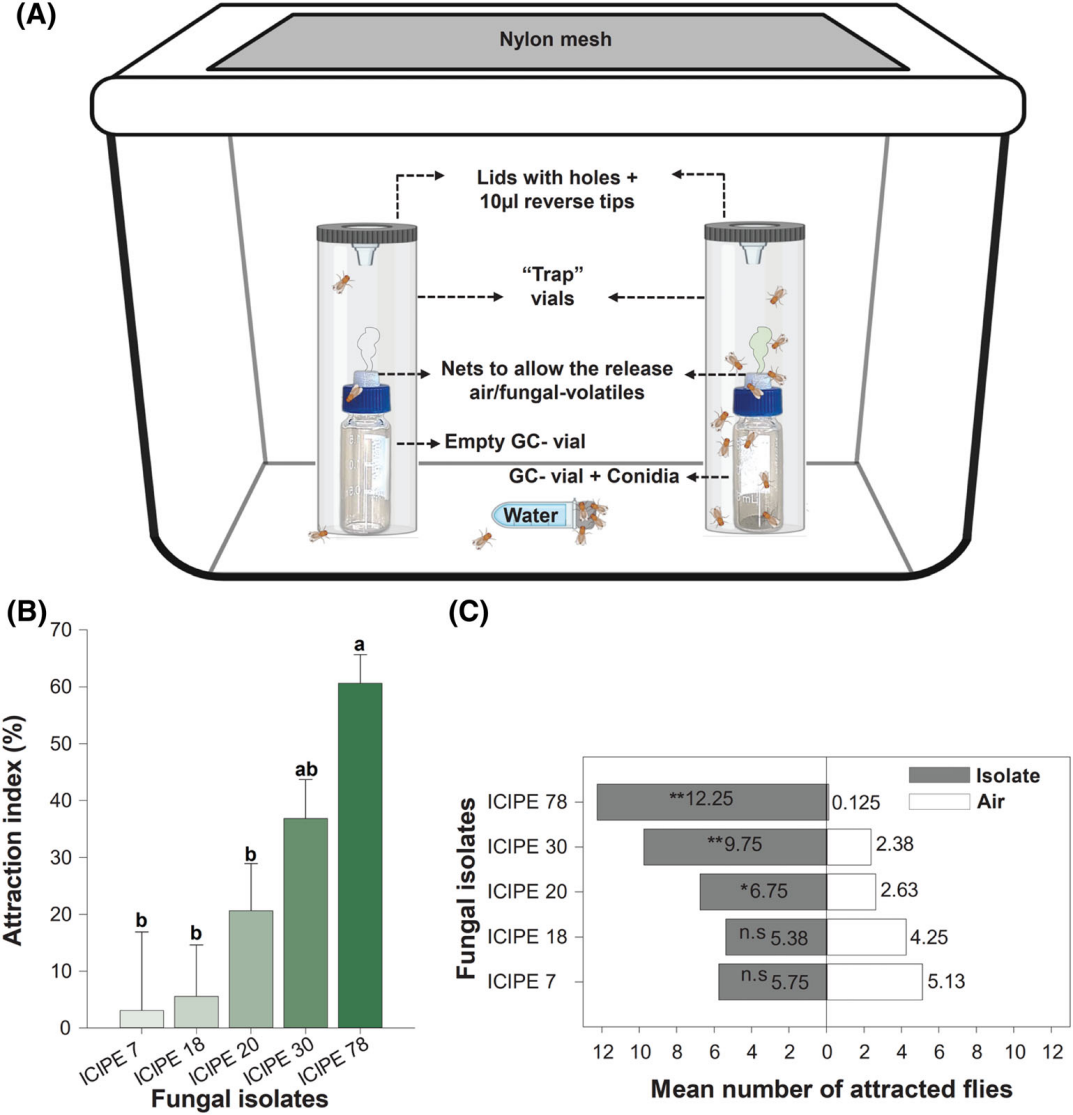
(A) Arena for the attraction assay of *Drosophila suzukii* to *Metarhizium anisopliae* conidia. (B) Attraction index (AI) of *D. suzukii* to fungal isolates after 24 h. (C) Mean number of attracted flies after 24 h. Different letters on the AI bars denote significant differences (Tukey HSD test at *α* = 0.05). Asterisks ‘*’ indicate significant differences (Wilcoxon signed‐rank paired, *: *P* ≤ 0.05, **: *P* ≤ 0.01, ***: *P* ≤ 0.001; n.s, not significant).

Overall, the five evaluated fungal isolates attracted *D. suzukii*, albeit the AI of the flies varied significantly among the isolates (*χ* ^2^(4) = 18 260, *P* < 0.001) (Fig. 4(B)). ICIPE 78 was the most attractive followed by ICIPE 30, which did not show a significant difference compared to ICIPE 20. Comparing the number of attracted flies of each isolate to clean air, ICIPE 78 had the highest number of attracted flies (*V* = 36, *P* = 0.0066), followed by ICIPE 30 (*V* = 36, *P* = 0.0070) and ICIPE 20 (*V* = 31, *P* = 0.0398). However, the number of flies attracted to ICIPE 7 and ICIPE 18 isolates was not significantly different from that of their respective controls (clean air) (Fig. 4(C)).

**Figure 5:**
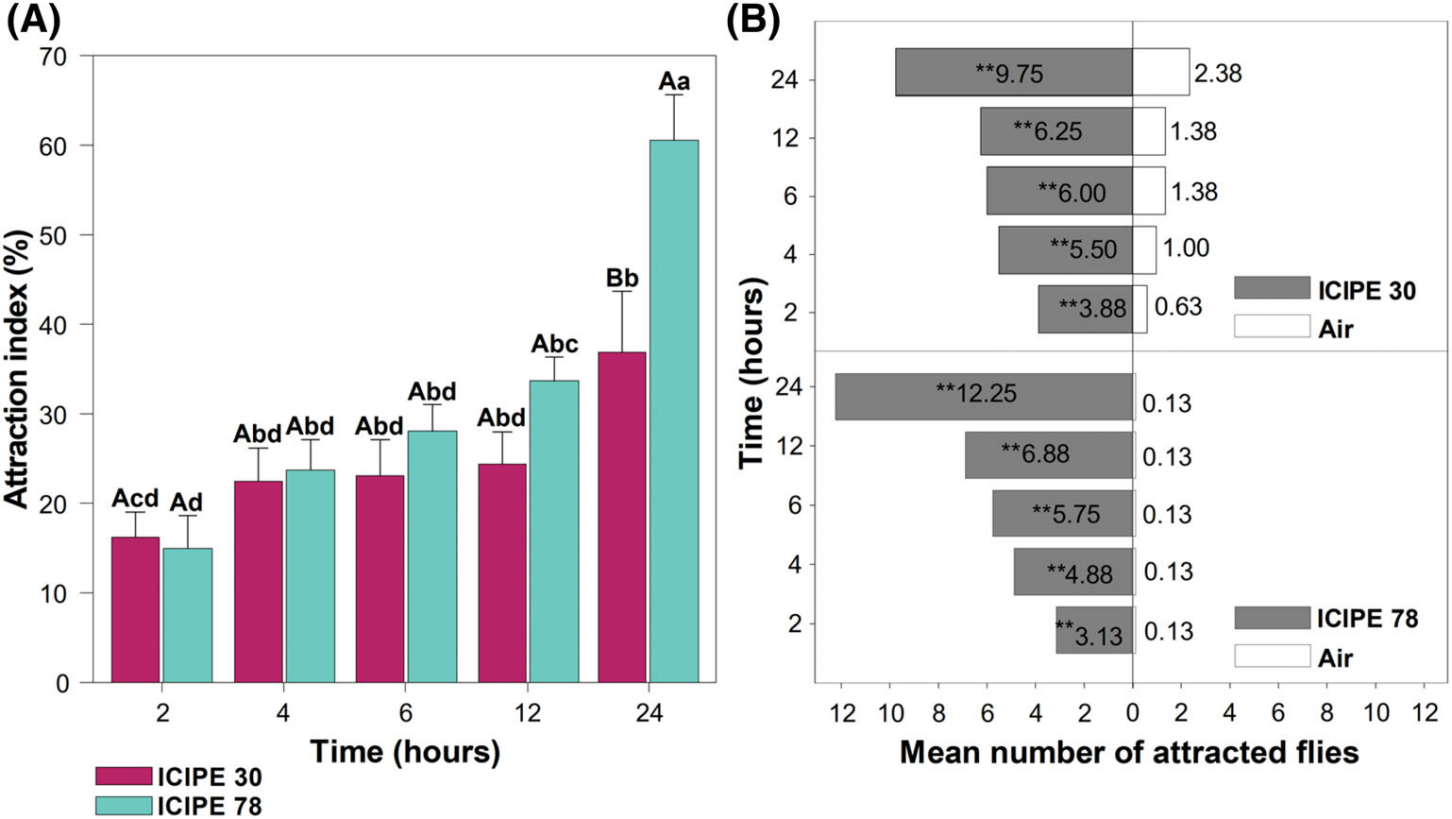
(A) Attraction index (AI) of *Drosophila suzukii* to *Metarhizium anisopliae* over 24 h. (B) Mean number of attracted flies over 24 h. Bars capped with the same lowercase and uppercase letters are not significantly different among time points and between isolates within the same time point, respectively (Tukey's HSD test, *α* = 0.05). Asterisks ‘*’ indicate significant differences (Wilcoxon signed‐rank paired, *: *P* ≤ 0.05, **: *P* ≤ 0.01, ***: *P* ≤ 0.001).

The subsequent evaluation of the most attractive isolates, ICIPE 30 and ICIPE 78, revealed a progressive increase in the flies' attraction over time (Fig. 5(A)). The AI to ICIPE 78 increased significantly over time (*χ* ^2^(9) = 12 533, *P* < 0.001) (Fig. 5(A)). When comparing the number of flies attracted to this isolate to that of the control (clean air) at different time points, the number of flies attracted to ICIPE 78 was significantly higher at all‐time points (*V* = 36, *P* = 0.0068; *V* = 36, *P* = 0.0064; *V* = 36, *P* = 0.0068; *V* = 36, *P* = 0.0063; and *V* = 36, *P* = 0.0066, at 2, 4, 6, 12 and 24 h, respectively). Similarly, the number of attracted flies to ICIPE 30 isolate compared to those attracted to the clean air was significantly higher at all‐time points (*V* = 36, *P* = 0.0065; *V* = 36, *P* = 0.0070; *V* = 36, *P* = 0.0070; *V* = 36, *P* = 0.0068; and *V* = 36, *P* = 0.0070 at, 2, 4, 6, 12 and 24 h, respectively) (Fig. 5(B)).

**Figure 6:**
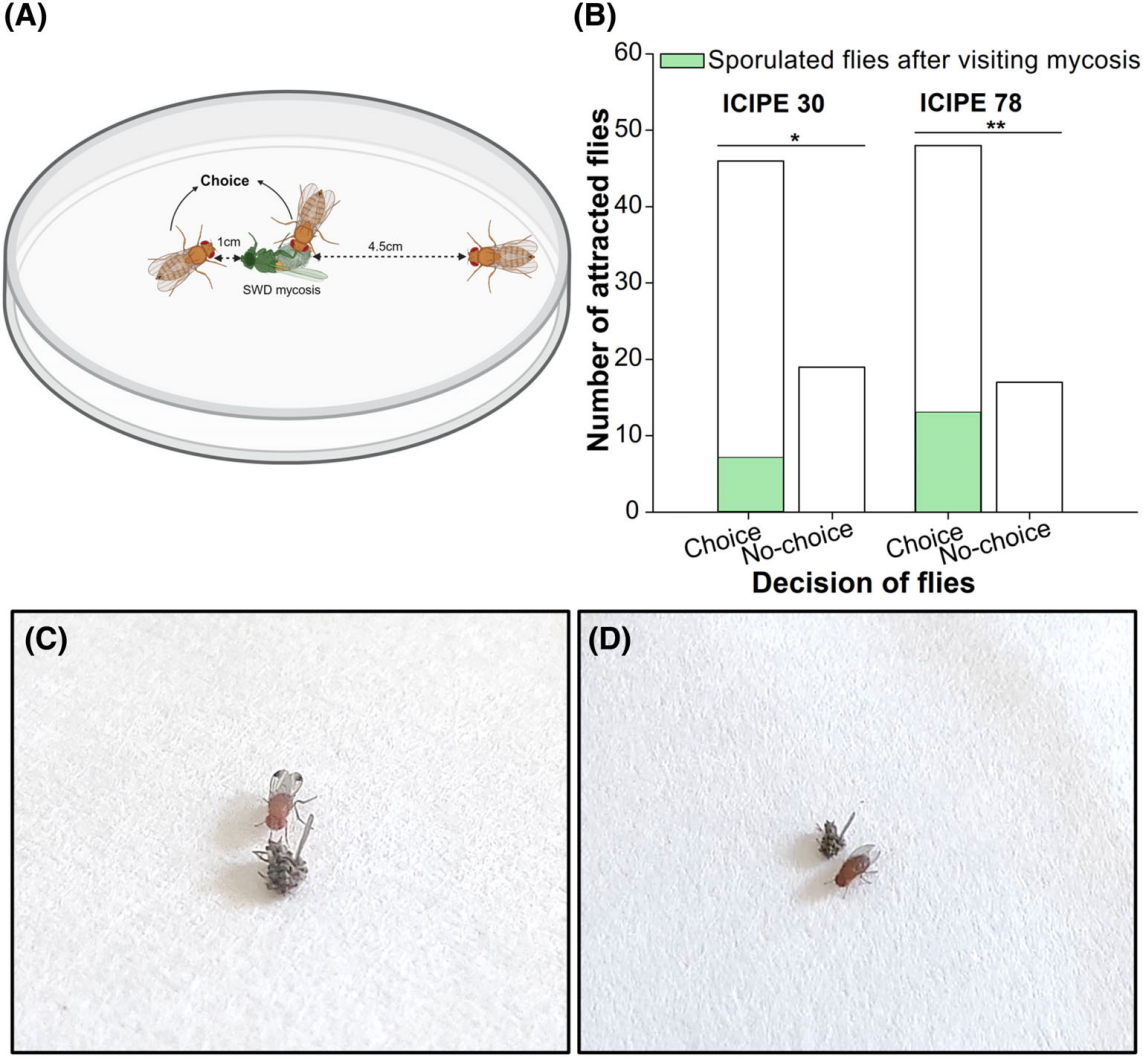
(A) Arena for the attraction assays of *Drosophila suzukii* to sporulated cadavers of *Metarhizium anisopliae*. (B) Number of flies that chose or did not choose sporulated cadavers. Asterisks ‘*’ indicate significant differences between the choice and no‐choice bars (*χ* ^2^ test, *: *P* < 0.033, **: *P* < 0.002). (C, D) Photographs of *D. suzukii* male and female attracted to insect mycosis, respectively.

In addition to the attraction response of the flies to fungi conidial volatiles from ICIPE 30 and ICIPE 78, *D. suzukii* sporulated cadavers attracted significantly more flies compared to the flies that did not make any choice (*χ* ^2^(1) = 5.024, *P* = 0.025 for ICIPE 30; and *χ* ^2^(1) = 6.859, *P* < 0.001 for ICIPE 78). Furthermore, some flies that were attracted to the sporulated cadavers became infected with the fungi (Fig. 6(B)).

## 4 DISCUSSION

Here, we identified five highly pathogenic *M. anisopliae* isolates through a pathogenicity screening of 20 fungal isolates of *M. anisopliae*, *M. brunneum*, *B. bassiana* and *P. isaria*. Two of these five isolates elicit significant attraction to adult *D. suzukii*. Attraction was demonstrated both to *M. anisopliae* dry conidia and towards sporulated cadavers. Furthermore, we showed horizontal transmission of the pathogen between flies, and that infection negatively affected fly fertility premortem.

This is the first study to evaluate Kenyan isolates of EPF against the recent invader, *D*. *suzukii*. Our finding of differential performance of different fungal isolates against *D*. *suzukii* is consistent with that reported by other authors.^29^, ^33^, ^34^, ^35^, ^36^, ^37^ For example, according Toledo‐Hernández *et al*.^29^ two *M. anisopliae* products (commercialized as Ma‐lu01 and Meta‐SIN®) yield mortality of about 90%, a finding which is in line with what we reported for the best performing isolates, ICIPE 7 and ICIPE 78. Also, Woltz *et al*.^35^ reported over 61% mortality of *D*. *suzukii* using the *M. anisopliae* F52. The same isolate was reported to cause over 80% mortality in *D. suzukii*.^33^

The pathogenicity of *M. anisopliae* ICIPE 7 and ICIPE 78 was further supported by previous findings against other pests. For instance, ICIPE 78 significantly reduced *Tetranychus urticae* Koch (Acari: Tetranychidae) populations in a screenhouse, with mite numbers nearly eliminated within 3 weeks.^38^ Also, ICIPE 7 caused 100% mortality in adult *S. frugiperda*.^11^ In a separate study, ICIPE 7 outperformed all the others evaluated isolates causing 93.7% mortality to the neonate larvae of *S. frugiperda*, while ICIPE 78, caused egg mortality of 87.0%.^12^ Fortunately, both isolates, have been commercialized (as Tickoff® and Mazao ACHIEVE® for ICIPE 7 and ICIPE 78, respectively) and widely used for management of other insect pests and vectors.^11^, ^12^ This should accelerate the extension of their labels and subsequent registration for use against *D. suzukii*.

In this study, we found that *D. suzukii* flies reared on the two media showed different mortality rates across all isolates. The higher mortality observed in flies reared on the artificial diet compared to those on raspberries might be due to the disruption of beneficial gut microbiota of the flies reared on the artificial diet, likely caused by the antibiotics added to the diet. Indeed, specific gut microbiota associated with *Drosophila*, such as *Acetobacter tropicalis*, help detoxify environmental toxins like the herbicide atrazine through a unique enzymatic pathway.^39^ This has been attributed to increased fly resilience to xenobiotic stress, effectively reducing the toxic effects of these compounds.

The results of horizontal transmission in this study suggest that flies inoculated with fungi can vector acquired conidia to the opposite sex during mating or any physical contact, thereby enhancing pest infection rates through autodissemination. Horizontal transmission of fungal *Metarhizium* has been demonstrated in *D. suzukii* and other related fruit flies. For instance, *D. suzukii* has been shown to transmit the fungus *M. brunneum* horizontally, thereby causing up to 48% and 24% mortality in male and female recipients, respectively.^40^ In other Diptera such as *Bactrocera cucurbitae* (Coquillett) and *Bactrocera zonata* (Saunders) (Diptera: Tephritidae), males infected with *M. anisopliae* successfully transmit the pathogen to healthy females, leading to high mortality rates.^41^ Similarly, the fungus was transmitted through direct contact between infected and healthy *Ceratitis capitata* Weid., *Ceratitis fasciventris* (Bezzi) and *Ceratitis cosyra* (Walker) (Diptera: Tephritidae), with conidia uptake by recipients ranging from 1.0 × 10^5^ to 2.5 × 10^5^.^42^ These studies strongly support the transmission of *M. anisopliae* found in the present study.

We also recorded a time‐dependent reduction in the number of fungal conidia following the infection by both donor and recipient flies. This suggests that flies actively remove conidia during hygienic grooming behavior. Indeed, this behavior has been documented in *Drosophila* following contact with fungal conidia. For example, *D. melanogaster* removed most *M. anisopliae* conidia from its body, while some were found trapped between the abdominal segments of the flies,^43^ a finding in line with this study, which found several thousand *M. anisopliae* conidia still retained by *D. suzukii* after 24 h. This highlights the high virulence of *M. anisopliae* ICIPE 7 and ICIPE 78 against *D. suzukii*. Also, when *D. melanogaster* was infected with another fungus, *B. bassiana*, the flies were able to clean conidia from almost all body parts using the same grooming behavior.^44^

Another finding linked to successful pathogen transmission was the negative impact of fungal isolates on parent fly fertility. While healthy parents produced an average of 10.08 offspring, this number dropped to 2.42 and 2.25 when parents were infected with ICIPE 7 and ICIPE 78, respectively. This further substantiates our finding of the high virulence of these isolates against *D*. *suzukii*. A similar effect of *M. anisopliae* on fecundity and fertility was reported in related flies. For example, Dimbi *et al*.^42^ reported a negative effect of *M. anisopliae* on the egg laying of *C. capitata*, *C. fasciventris* and *C. cosyra* with a fecundity reduction of 82%, 73%, and 37%, respectively. In another study, *C. capitata* infected with *M. anisopliae* suffered a significant reduction in fecundity and fertility at 6 days premortem, ranging from 58.4% to 72.1% and 28.6% to 45.9%, respectively.^45^ In addition, female *B. cucurbitae* produced significantly fewer eggs (15 or fewer eggs) compared to untreated females (30 eggs) when infected with *M. anisopliae*, regardless of whether the female or male was the donor.^41^

For EPF to be effective against a target pest, the fungi must come into contact with the pest. However, some pests such as termites can detect *M. anisopliae* conidia (including ICIPE 30) via olfaction, allowing them to avoid physical contact with the fungus.^46^, ^47^ Moreover, the fungus *Cordyceps fumosorosea* FG340 repelled the beet armyworm *Spodoptera exigua* (Hübner) (Lepidoptera: Noctuidae).^48^ Interestingly, in this study, we found that the conidia of *M. anisopliae* isolates attracted healthy *D. suzukii* flies. Furthermore, for ICIPE 30 and ICIPE 78, we demonstrated that sporulated cadavers of *D. suzukii* attracted and infected healthy flies. Similar EPF attraction behavior was shown in *Anopheles stephensi* towards *B. bassiana* and *M. anisopliae* conidia, but the exact mechanisms driving this behavior remain unclear.^20^ Likewise, in the present study, the mechanisms underlying *D. suzukii* attraction to *M. anisopliae* isolates remain unknown and warrant further exploration.

Volatile cues are likely involved in the *D. suzukii*–*M. anisopliae* interaction. Indeed, EPF have been reported to produce volatile compounds that can attract insects. For instance, *B. bassiana* can attract the green peach aphid through volatile emissions.^21^ Similarly, EPF of the genus *Lecanicillium* emit volatile compounds (such as acetic acid) that elicit attraction behavior in western flower thrips.^49^ The attraction of *D. suzukii* to *M. anisopliae* used in this study may be due to the release of volatile compounds that serve as food cues to the pre‐starved *D. suzukii*, considering that *Drosophila* species feed on microorganisms.^50^, ^51^ *Metarhizium anisopliae* is primarily a soilborne fungus and depends on vectors for dispersal, which could be facilitated through behavioral manipulation of *D. suzukii*. Active dispersal mechanisms enhance the transmission of some EPF.^52^ For example, grasshoppers infected with *Entomophaga grylli* (Fresenius) Batko, climb to elevated positions on vegetation just before death, enhancing conidia dispersal and pathogen transmission.^53^ Another example is that of *Entomophthora muscae* (*Cohn*) Fresen, which alters the chemical profile of infected female housefly cadavers to attract healthy males for fatal matings.^54^ Such fungal manipulation of hosts may explain the attraction of *D. suzukii* to *M. anisopliae* reported in the current study.

Based on this study, it can be concluded that two *M. anisopliae* isolates, ICIPE 7 and ICIPE 78, are the most effective against *D*. *suzukii* in terms of mortality, horizontal transmission, and fertility effects. Therefore, these isolates are excellent candidates for development as biopesticides within the context of IPM of this pest. Since these isolates have already been commercialized as biopesticides against other pests, this could simplify and accelerate their registration for use against *D. suzukii* through a label extension. The novel finding that ICIPE 78 is attracted to *D*. *suzukii* could be further explored to enhance pest suppression. For example, targeted spraying of this fungal product could sustainably reduce the pest populations, especially since the dual action attract‐and‐kill of ICIPE 78 can improve IPM strategies as self‐disseminating biocontrol agents. While these findings provide a foundation for microbial control of *D*. *suzukii*, further research is recommended to identify the compounds responsible for the attraction and to explore potential applications of these isolates for monitoring and overall pest management.

## CONFLICT OF INTEREST

All authors declare no conflicts of interest.

## Supporting information


**Figure S1.** Screening of fungal isolates of *Metarhizium anisopliae*, *Beauveria bassiana* and *Paecilomyces isaria* and *Metarhizium brunneum* against *Drosophila suzukii* adults after 10 days monitoring. Bars capped with the same lowercase letters are not significantly different among fungal isolates (Tukey's HSD test, *α* = 0.05).
**Figure S2.** Acquisition of *Metarhizium anisopliae* dry conidia and horizontal transmission by *Drosophila suzukii* adults. The asterisk symbol ‘*’ indicate significant differences among/between means (*: *P* ≤ 0.05; n.s, not significant). Don., donor; Recip., recipient.
**Table S1.** Survival analysis of *Drosophila suzukii* groups exposed to *Metarhizium anisopliae* isolates. Log‐rank test comparisons between treatment and control.
**Table S2.** Generalized linear model results for multiple pairwise comparisons of mean mortality in *Drosophila suzukii* treated with *Metarhizium anisopliae* (Tukey HSD test).
**Table S3.** Results of the Wilcoxon signed‐rank paired test comparing the number of *Metarhizium anisopliae* dry conidia retained by donor *versus* recipient‐flies following horizontal transmission.
**Table S4.** Results of Kruskal–Wallis tests comparing mean number of offspring from daily oviposition by infected and non‐infected fly parents.

## Data Availability

The data that support the findings of this study are available from the corresponding author upon reasonable request.
