# Supplementary material for: Conned by the enemy: the entomopathogenic fungus Metarhizium anisopliae lures and kills Drosophila suzukii
Source: Pest Manag Sci. 2026 Jan 27;82(5):4595–606. doi: 10.1002/ps.70576 (PMC13071260; doi:10.1002/ps.70576)
Supplement: Supplementary file 1 — Figure S1. Screening of fungal isolates of Metarhizium anisopliae, Beauveria bassiana and Paecilomyces isaria and Metarhizium brunneum against Drosophila suzukii adults after 10 days monitoring. Bars capped with the same lowercase letters are not significantly different among fungal isolates (Tukey's HSD test, α = 0.05). Figure S2. Acquisition of Metarhizium anisopliae dry conidia and horizontal transmission by Drosophila suzukii adults. The asterisk symbol ‘*’ indicate significant differences among/between means (*: P ≤ 0.05; n.s, not significant). Don., donor; Recip., recipient. Table S1. Survival analysis of Drosophila suzukii groups exposed to Metarhizium anisopliae isolates. Log‐rank test comparisons between treatment and control. Table S2. Generalized linear model results for multiple pairwise comparisons of mean mortality in Drosophila suzukii treated with Metarhizium anisopliae (Tukey HSD test). Table S3. Results of the Wilcoxon signed‐rank paired test comparing the number of Metarhizium anisopliae dry conidia retained by donor versus recipient‐flies following horizontal transmission. Table S4. Results of Kruskal–Wallis tests comparing mean number of offspring from daily oviposition by infected and non‐infected fly parents. [file PS-82-4595-s001.docx]

**SUPPLEMENTARY DOCUMENT**


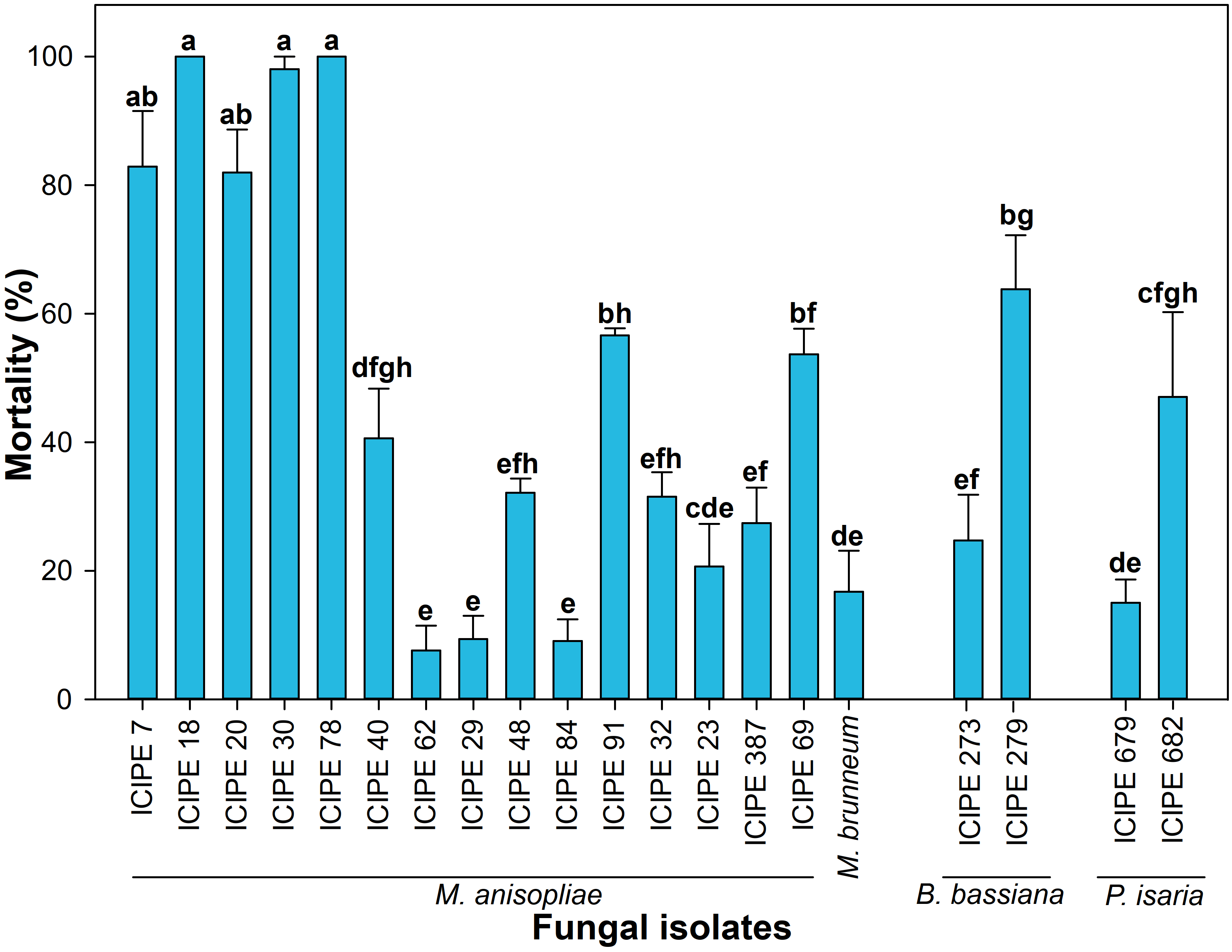
**Figure S1.** Screening of fungal isolates of *Metarhizium anisopliae*, *Beauveria bassiana* and *Paecilomyces isaria* and *M. brunneum* against *Drosophila suzukii* adults after 10 days monitoring. Bars capped with the same lowercase letters are not significantly different among fungal isolates (Tukey’s HSD test, α = 0.05).


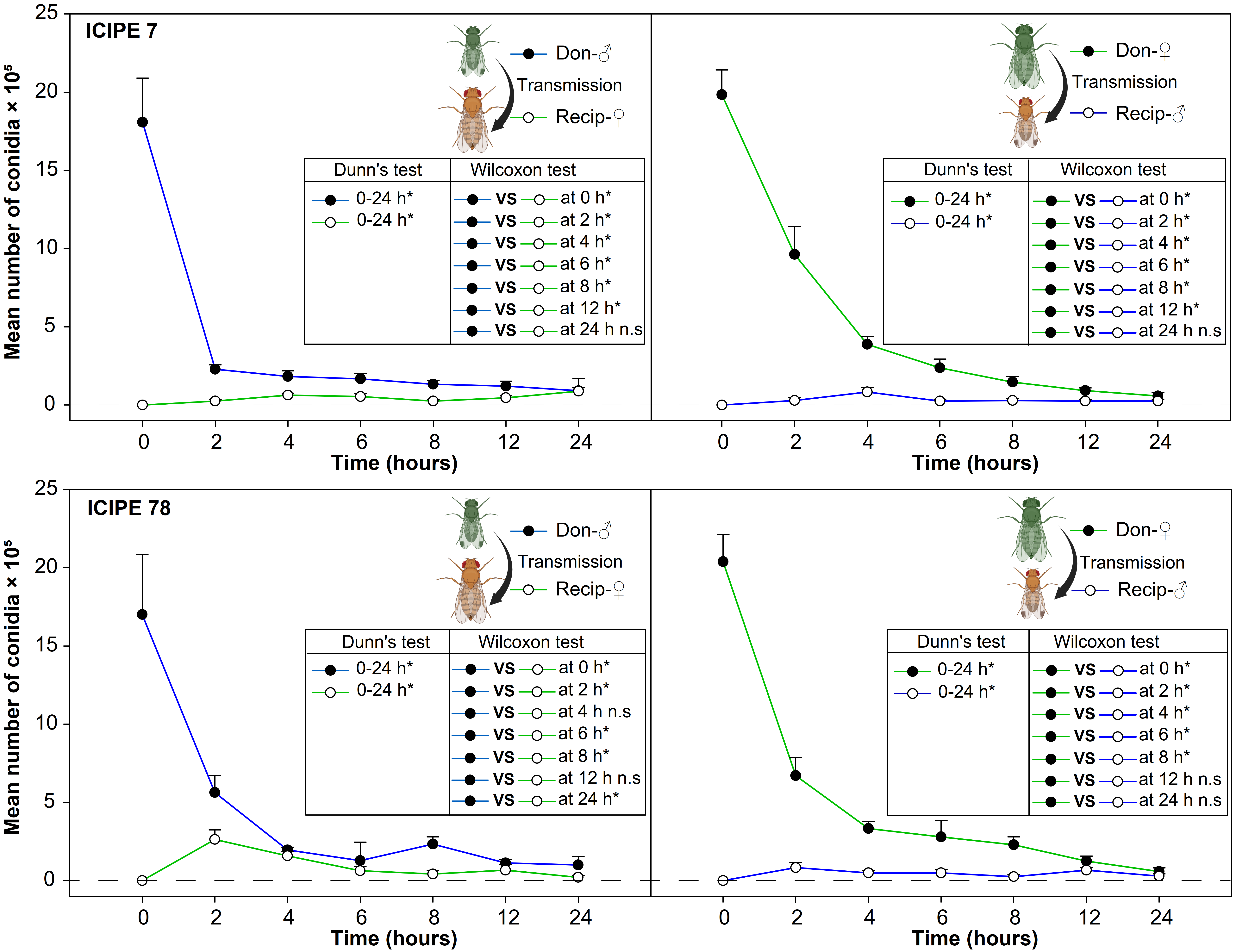
**Figure S2.** Acquisition of *Metarhizium anisopliae* dry conidia and horizontal transmission by *Drosophila suzukii* adults. The asterisk symbol “*” indicate significant differences among/between means (* *p* ≤ 0.05; n.s = not significant). Abbreviations: Don. = donor; Recip. = recipient.

**Table S1.** Survival analysis of *Drosophila suzukii* groups exposed to *Metarhizium anisopliae* isolates. Log-rank test comparisons between treatment and control.

| **Group** | **N** | **Observed** | **Expected** | **(O-E)²/E** | **χ² (df=1)** | **p-value** |
| --- | --- | --- | --- | --- | --- | --- |
| ***Artificial diet reared flies*** |  |  |  |  |  |  |
| Ctrl | 126 | 2 | 33 | 29.1 | 64.4 | **< 0.001 (***)** |
| ICIPE 7 | 126 | 64 | 33 | 29.1 |  |  |
| Ctrl | 126 | 2 | 31 | 27.1 | 61.3 | **< 0.001 (***)** |
| ICIPE 18 | 126 | 60 | 31 | 27.1 |  |  |
| Ctrl | 126 | 2 | 27 | 23.1 | 57.8 | **< 0.001 (***)** |
| ICIPE 20 | 126 | 52 | 27 | 23.1 |  |  |
| Ctrl | 126 | 2 | 32.5 | 28.6 | 67.2 | **< 0.001 (***)** |
| ICIPE 30 | 126 | 63 | 32.5 | 28.6 |  |  |
| Ctrl | 126 | 2 | 31 | 27.1 | 58.3 | **< 0.001 (***)** |
| ICIPE 78 | 126 | 60 | 31 | 27.1 |  |  |
| ***Raspberry fruit reared flies*** |  |  |  |  |  |  |
| Ctrl | 33 | 2 | 11.3 | 7.65 | 11.5 | **0.0007 (***)** |
| ICIPE 7 | 93 | 54 | 44.7 | 1.93 |  |  |
| Ctrl | 33 | 2 | 8.53 | 5 | 7.7 | **0.006 (**)** |
| ICIPE 18 | 93 | 40 | 33.47 | 1.27 |  |  |
| Ctrl | 33 | 2 | 6.89 | 3.47 | 4.9 | **0.03 (*)** |
| ICIPE 20 | 93 | 32 | 27.11 | 0.882 |  |  |
| Ctrl | 33 | 2 | 7.15 | 3.706 | 6.2 | **0.01 (*)** |
| ICIPE 30 | 93 | 33 | 27.85 | 0.951 |  |  |
| Ctrl | 33 | 2 | 12.3 | 8.62 | 12.9 | **0.0003 (***)** |
| ICIPE 78 | 93 | 60 | 49.7 | 2.13 |  |  |

Ctrl = Control.

**Table S2.** Generalized linear model results for multiple pairwise comparisons of mean mortality in *Drosophila suzukii* treated with *Metarhizium anisopliae* (Tukey HSD test).

| **Comparison** | **Estimate** | **Std. Error** | **z-value** | **p-value** |
| --- | --- | --- | --- | --- |
| Ctrl DF - Ctrl RF == 0 | 6.11E-14 | 5.81E+00 | 0 | 1 |
| ICIPE 7 RF - Ctrl RF == 0 | 8.61E+01 | 5.81E+00 | 14.838 | <0.01 *** |
| ICIPE 7 DF - Ctrl RF == 0 | 9.67E+01 | 5.81E+00 | 16.651 | <0.01 *** |
| ICIPE 18 RF - Ctrl RF == 0 | 6.20E+01 | 5.81E+00 | 10.683 | <0.01 *** |
| ICIPE 18 DF - Ctrl RF == 0 | 9.67E+01 | 5.81E+00 | 16.651 | <0.01 *** |
| ICIPE 20 RF - Ctrl RF == 0 | 4.83E+01 | 5.81E+00 | 8.326 | <0.01 *** |
| ICIPE 20 DF - Ctrl RF == 0 | 8.07E+01 | 5.81E+00 | 13.908 | <0.01 *** |
| ICIPE 30 RF - Ctrl RF == 0 | 5.01E+01 | 5.81E+00 | 8.628 | <0.01 *** |
| ICIPE 30 DF - Ctrl RF == 0 | 9.33E+01 | 5.81E+00 | 16.077 | <0.01 *** |
| ICIPE 78 RF - Ctrl RF == 0 | 9.67E+01 | 5.81E+00 | 16.651 | <0.01 *** |
| ICIPE 78 DF - Ctrl RF == 0 | 9.67E+01 | 5.81E+00 | 16.651 | <0.01 *** |
| ICIPE 7 RF - Ctrl DF == 0 | 8.61E+01 | 5.81E+00 | 14.838 | <0.01 *** |
| ICIPE 7 DF - Ctrl DF == 0 | 9.67E+01 | 5.81E+00 | 16.651 | <0.01 *** |
| ICIPE 18 RF - Ctrl DF == 0 | 6.20E+01 | 5.81E+00 | 10.683 | <0.01 *** |
| ICIPE 18 DF - Ctrl DF == 0 | 9.67E+01 | 5.81E+00 | 16.651 | <0.01 *** |
| ICIPE 20 RF - Ctrl DF == 0 | 4.83E+01 | 5.81E+00 | 8.326 | <0.01 *** |
| ICIPE 20 DF - Ctrl DF == 0 | 8.07E+01 | 5.81E+00 | 13.908 | <0.01 *** |
| ICIPE 30 RF - Ctrl DF == 0 | 5.01E+01 | 5.81E+00 | 8.628 | <0.01 *** |
| ICIPE 30 DF - Ctrl DF == 0 | 9.33E+01 | 5.81E+00 | 16.077 | <0.01 *** |
| ICIPE 78 RF - Ctrl DF == 0 | 9.67E+01 | 5.81E+00 | 16.651 | <0.01 *** |
| ICIPE 78 DF - Ctrl DF == 0 | 9.67E+01 | 5.81E+00 | 16.651 | <0.01 *** |
| ICIPE 7 DF - ICIPE 7 RF == 0 | 1.05E+01 | 5.81E+00 | 1.813 | 0.8111 |
| ICIPE 18 RF - ICIPE 7 RF == 0 | -2.41E+01 | 5.81E+00 | -4.155 | <0.01 *** |
| ICIPE 18 DF - ICIPE 7 RF == 0 | 1.05E+01 | 5.81E+00 | 1.813 | 0.811 |
| ICIPE 20 RF - ICIPE 7 RF == 0 | -3.78E+01 | 5.81E+00 | -6.512 | <0.01 *** |
| ICIPE 20 DF - ICIPE 7 RF == 0 | -5.40E+00 | 5.81E+00 | -0.93 | 0.9988 |
| ICIPE 30 RF - ICIPE 7 RF == 0 | -3.61E+01 | 5.81E+00 | -6.21 | <0.01 *** |
| ICIPE 30 DF - ICIPE 7 RF == 0 | 7.19E+00 | 5.81E+00 | 1.239 | 0.9859 |
| ICIPE 78 RF - ICIPE 7 RF == 0 | 1.05E+01 | 5.81E+00 | 1.813 | 0.8113 |
| ICIPE 78 DF - ICIPE 7 RF == 0 | 1.05E+01 | 5.81E+00 | 1.813 | 0.8114 |
| ICIPE 18 RF - ICIPE 7 DF == 0 | -3.47E+01 | 5.81E+00 | -5.968 | <0.01 *** |
| ICIPE 18 DF - ICIPE 7 DF == 0 | -1.42E-14 | 5.81E+00 | 0 | 1 |
| ICIPE 20 RF - ICIPE 7 DF == 0 | -4.83E+01 | 5.81E+00 | -8.326 | <0.01 *** |
| ICIPE 20 DF - ICIPE 7 DF == 0 | -1.59E+01 | 5.81E+00 | -2.743 | 0.2054 |
| ICIPE 30 RF - ICIPE 7 DF == 0 | -4.66E+01 | 5.81E+00 | -8.023 | <0.01 *** |
| ICIPE 30 DF - ICIPE 7 DF == 0 | -3.33E+00 | 5.81E+00 | -0.574 | 1 |
| ICIPE 78 RF - ICIPE 7 DF == 0 | 1.42E-14 | 5.81E+00 | 0 | 1 |
| ICIPE 78 DF - ICIPE 7 DF == 0 | 1.42E-14 | 5.81E+00 | 0 | 1 |
| ICIPE 18 DF - ICIPE 18 RF == 0 | 3.47E+01 | 5.81E+00 | 5.968 | <0.01 *** |
| ICIPE 20 RF - ICIPE 18 RF == 0 | -1.37E+01 | 5.81E+00 | -2.357 | 0.4355 |
| ICIPE 20 DF - ICIPE 18 RF == 0 | 1.87E+01 | 5.81E+00 | 3.225 | 0.0569 |
| ICIPE 30 RF - ICIPE 18 RF == 0 | -1.19E+01 | 5.81E+00 | -2.055 | 0.6551 |
| ICIPE 30 DF - ICIPE 18 RF == 0 | 3.13E+01 | 5.81E+00 | 5.394 | <0.01 *** |
| ICIPE 78 RF - ICIPE 18 RF == 0 | 3.47E+01 | 5.81E+00 | 5.968 | <0.01 *** |
| ICIPE 78 DF - ICIPE 18 RF == 0 | 3.47E+01 | 5.81E+00 | 5.968 | <0.01 *** |
| ICIPE 20 RF - ICIPE 18 DF == 0 | -4.83E+01 | 5.81E+00 | -8.326 | <0.01 *** |
| ICIPE 20 DF - ICIPE 18 DF == 0 | -1.59E+01 | 5.81E+00 | -2.743 | 0.2051 |
| ICIPE 30 RF - ICIPE 18 DF == 0 | -4.66E+01 | 5.81E+00 | -8.023 | <0.01 *** |
| ICIPE 30 DF - ICIPE 18 DF == 0 | -3.33E+00 | 5.81E+00 | -0.574 | 1 |
| ICIPE 78 RF - ICIPE 18 DF == 0 | 2.84E-14 | 5.81E+00 | 0 | 1 |
| ICIPE 78 DF - ICIPE 18 DF == 0 | 2.84E-14 | 5.81E+00 | 0 | 1 |
| ICIPE 20 DF - ICIPE 20 RF == 0 | 3.24E+01 | 5.81E+00 | 5.582 | <0.01 *** |
| ICIPE 30 RF - ICIPE 20 RF == 0 | 1.75E+00 | 5.81E+00 | 0.302 | 1 |
| ICIPE 30 DF - ICIPE 20 RF == 0 | 4.50E+01 | 5.81E+00 | 7.751 | <0.01 *** |
| ICIPE 78 RF - ICIPE 20 RF == 0 | 4.83E+01 | 5.81E+00 | 8.326 | <0.01 *** |
| ICIPE 78 DF - ICIPE 20 RF == 0 | 4.83E+01 | 5.81E+00 | 8.326 | <0.01 *** |
| ICIPE 30 RF - ICIPE 20 DF == 0 | -3.07E+01 | 5.81E+00 | -5.28 | <0.01 *** |
| ICIPE 30 DF - ICIPE 20 DF == 0 | 1.26E+01 | 5.81E+00 | 2.169 | 0.5721 |
| ICIPE 78 RF - ICIPE 20 DF == 0 | 1.59E+01 | 5.81E+00 | 2.743 | 0.2054 |
| ICIPE 78 DF - ICIPE 20 DF == 0 | 1.59E+01 | 5.81E+00 | 2.743 | 0.2046 |
| ICIPE 30 DF - ICIPE 30 RF == 0 | 4.33E+01 | 5.81E+00 | 7.449 | <0.01 *** |
| ICIPE 78 RF - ICIPE 30 RF == 0 | 4.66E+01 | 5.81E+00 | 8.023 | <0.01 *** |
| ICIPE 78 DF - ICIPE 30 RF == 0 | 4.66E+01 | 5.81E+00 | 8.023 | <0.01 *** |
| ICIPE 78 RF - ICIPE 30 DF == 0 | 3.33E+00 | 5.81E+00 | 0.574 | 1 |
| ICIPE 78 DF - ICIPE 30 DF == 0 | 3.33E+00 | 5.81E+00 | 0.574 | 1 |
| ICIPE 78 DF - ICIPE 78 RF == 0 | 0.00E+00 | 5.81E+00 | 0 | 1 |

DF = Diet reared flies, RF = Raspberry fruit reared flies and Ctrl = Control.

| **Isolate** | **Time Point** | **V statistic** | **p-value** | **Significant (α=0.05)** |
| --- | --- | --- | --- | --- |
| **ICIPE 7** |  |  |  |  |
| Donor-♂ VS Recipient-♀ | 0 h | 21 | 0.01563 | Yes |
|  | 2 h | 21 | 0.01776 | Yes |
|  | 4 h | 15 | 0.02895 | Yes |
|  | 6 h | 20 | 0.02924 | Yes |
|  | 8 h | 21 | 0.01552 | Yes |
|  | 12 h | 10 | 0.04876 | Yes |
|  | 24 h | 10 | 0.2948 | No |
| Donor-♀ VS Recipient-♂ | 0 h | 21 | 0.01776 | Yes |
|  | 2 h | 21 | 0.01563 | Yes |
|  | 4 h | 21 | 0.01563 | Yes |
|  | 6 h | 20 | 0.03125 | Yes |
|  | 8 h | 21 | 0.01776 | Yes |
|  | 12 h | 21 | 0.01776 | Yes |
|  | 24 h | 11.5 | 0.1704 | No |
| **ICIPE 78** |  |  |  |  |
| Donor-♂ VS Recipient-♀ | 0 h | 21 | 0.01563 | Yes |
|  | 2 h | 20 | 0.02924 | Yes |
|  | 4 h | 6 | 0.09072 | No |
|  | 6 h | 20 | 0.02924 | Yes |
|  | 8 h | 21 | 0.01776 | Yes |
|  | 12 h | 16 | 0.1563 | No |
|  | 24 h | 10 | 0.04876 | Yes |
| Donor-♀ VS Recipient-♂ | 0h | 21 | 0.01563 | Yes |
|  | 2h | 21 | 0.01563 | Yes |
|  | 4h | 21 | 0.01776 | Yes |
|  | 6h | 20 | 0.03125 | Yes |
|  | 8h | 21 | 0.01776 | Yes |
|  | 12h | 12 | 0.1359 | No |
|  | 24h | 10.5 | 0.2491 | No |

**Table S3**. Results of the Wilcoxon signed-rank paired test comparing the number of *Metarhizium anisopliae* dry conidia retained by donor vs. recipient-flies following horizontal transmission.

**Table S4**. Results of Kruskal-Wallis tests comparing mean number of offspring from daily oviposition by infected and non-infected fly parents.

| **Isolate** | **Variable** | **χ²** | **df** | **p-value** | **Significant Comp. (Dunn's test)** |
| --- | --- | --- | --- | --- | --- |
| **ICIPE 7** | **Comp. within each group** |  |  |  |  |
|  | Control | 12.855 | 9 | 0.169 | Not significant |
|  | Don-♀+Recip-♂ | 52.001 | 9 | 4.52E-08 | Day10 < Day1, Day2, Day4, Day5, Day6; Day2 > Day3-Day9 |
|  | Don-♂+Recip-♀ | 30.456 | 9 | 0.00037 | Day10, Day9 < Day2, Day3; Day2 > Day10, Day9 |
|  | Don-♂+Don-♀ | 38.976 | 9 | 1.16E-05 | Day10, Day5-Day9 < Day2, Day3; Day2 > Day10, Day5-Day9 |
|  | **Comp. across groups** |  |  |  |  |
|  | Day1 | 20.278 | 3 | 1.49E-04 | Don-♂+Don-♀ < Control (p=0.0001); Don-♀+Recip-♂ < Control (p=0.0128); Don-♂+Recip-♀ < Control (p=0.0010) |
|  | Day2 | 9.799 | 3 | 2.04E-02 | Don-♂+Don-♀ < Control (p=0.0080) |
|  | Day3 | 12.196 | 3 | 6.74E-03 | Don-♂+Don-♀ < Control (p=0.0077); Don-♀+Recip-♂ < Control (p=0.0074) |
|  | Day4 | 30.339 | 3 | 1.17E-06 | Don-♂+Don-♀ < Control (p<0.0001); Don-♀+Recip-♂ < Control (p<0.0001); Don-♂+Recip-♀ < Control (p=0.0117) |
|  | Day5 | 30.089 | 3 | 1.32E-06 | Don-♂+Don-♀ < Control (p<0.0001); Don-♀+Recip-♂ < Control (p<0.0001); Don-♂+Recip-♀ < Control (p=0.0014) |
|  | Day6 | 33.952 | 3 | 2.03E-07 | Don-♂+Don-♀ < Control (p<0.0001); Don-♀+Recip-♂ < Control (p<0.0001); Don-♂+Recip-♀ < Control (p=0.0002) |
|  | Day7 | 41.843 | 3 | 4.33E-09 | Control > all treatments (p<0.0001) |
|  | Day8 | 42.812 | 3 | 2.70E-09 | Control > all treatments (p<0.0001) |
|  | Day9 | 45.747 | 3 | 6.42E-10 | Control > all treatments (p<0.0001) |
|  | Day10 | 45.76 | 3 | 6.38E-10 | Control > all treatments (p<0.0001) |
| **ICIPE 78** | **Comp. within each group** |  |  |  |  |
|  | Control | 12.855 | 9 | 0.169 | Not significant |
|  | Don-♀+Recip-♂ | 60.078 | 9 | 1.30E-09 | Day10 < Day1, Day2, Day4-Day6; Day2 > Day3-Day9 |
|  | Don-♂+Recip-♀ | 36.7 | 9 | 2.98E-05 | Day10, Day9 < Day1, Day2; Day2 > Day10, Day9 |
|  | Don-♂+Don-♀ | 41.784 | 9 | 3.60E-06 | Day10, Day7-Day9 < Day2, Day3; Day2 > Day10, Day7-Day9 |
|  | **Comp. across groups** |  |  |  |  |
|  | Day1 | 22.707 | 3 | 4.65E-05 | Don-♂+Don-♀ < Control; Don-♀+Recip-♂ < Control; Don-♂+Don-♀ vs Don-♂+Recip-♀ (p=0.0946) |
|  | Day2 | 10.856 | 3 | 0.01253 | Control > Don-♂+Don-♀ (p=0.0054); Control > Don-♀+Recip-♂ (p=0.0434) |
|  | Day3 | 15.243 | 3 | 0.001621 | Control > Don-♂+Don-♀ (p=0.0190); Control > Don-♀+Recip-♂ (p=0.0007); Control >Don-♂+Recip-♀ (p=0.0181) |
|  | Day4 | 30.976 | 3 | 8.60E-07 | Don-♂+Don-♀ < Control (p<0.0001); Don-♀+Recip-♂ < Control (p<0.0001); Don-♂+Recip-♀ < Control (p=0.0012) |
|  | Day5 | 33.711 | 3 | 2.28E-07 | Don-♂+Don-♀ < Control (p<0.0001); Don-♀+Recip-♂ < Control (p<0.0001); Don-♂+Recip-♀ < Control (p=0.0005) |
|  | Day6 | 34.787 | 3 | 1.35E-07 | Don-♂+Don-♀ < Control (p<0.0001); Don-♀+Recip-♂ < Control (p<0.0001); Don-♂+Recip-♀ < Control (p=0.0005) |
|  | Day7 | 36.775 | 3 | 5.14E-08 | Don-♂+Don-♀ < Control (p<0.0001); Don-♀+Recip-♂ < Control (p<0.0001); Don-♂+Recip-♀ < Control (p=0.0002) |
|  | Day8 | 41.117 | 3 | 6.18E-09 | Don-♂+Don-♀ < Control (p<0.0001); Don-♀+Recip-♂ < Control (p<0.0001); Don-♂+Recip-♀ < Control (p<0.0001) |
|  | Day9 | 45.747 | 3 | 6.42E-10 | Don-♂+Don-♀ < Control (p<0.0001); Don-♀+Recip-♂ < Control (p<0.0001); Don-♂+Recip-♀ < Control (p<0.0001) |
|  | Day10 | 45.76 | 3 | 6.38E-10 | Control > all treatments (p<0.0001) |

Abbreviations: Don. = donor; Recip. = recipient.
